# Advanced Percutaneous Endovascular Techniques for the Treatment of Acute Bowel Ischemia—Retrograde Endovascular Recanalization and Lithotripsy-Assisted Angioplasty: Case Report

**DOI:** 10.3390/jcm14093014

**Published:** 2025-04-27

**Authors:** Paweł Latacz, Tadeusz Popiela, Monika Stępień, Marian Simka

**Affiliations:** 1 Department of Radiology, Jagiellonian University Medical College, 43-007 Krakow, Poland; pawel.latacz@uj.edu.pl (P.L.); tadeusz.popiela@uj.edu.pl (T.P.); monika.1.stepien@uj.edu.pl (M.S.); 2 Department of Vascular Surgery and Angiology, Brothers of Mercy St. John Grande’s Hospital, 31-061 Krakow, Poland; 3 Department of Anatomy, University of Opole, 45-040 Opole, Poland

**Keywords:** acute bowel ischemia, mesenteric artery occlusion, mesenteric artery stenting, retrograde revascularization, lithotripsy-assisted angioplasty

## Abstract

**Background:** Acute bowel ischemia that develops secondarily to thrombotic occlusion of the superior mesenteric artery is a life-threatening abdominal emergency. Although an open surgical repair is still the main treatment modality for this pathology, percutaneous endovascular revascularization is currently recognized as an alternative therapeutic option. However, in some patients, endovascular repair of the occluded superior mesenteric artery is technically very challenging. **Case description:** We provide technical details of percutaneous endovascular revascularization of the superior mesenteric artery in a patient presenting with highly calcified plaques extending to the aortic wall, with an associated risk of the aortic wall rupturing after standard balloon angioplasty. The patient was managed using the lithotripsy-assisted angioplasty, in order to minimize the risk of aortic injury. **Conclusion:** During endovascular reconstruction of the superior mesenteric artery for acute bowel ischemia, on the one hand, a full revascularization of the ischemic bowel should be achieved. On the other hand, the procedure should not be too aggressive. In this technical note, we demonstrated that even very difficult cases can be successfully managed endovascularly, if a tailored approach is used and proper endovascular devices are applied.

## 1. Introduction

Although visceral arteries are quite often affected by atherosclerosis, acute bowel ischemia (ABI) that develops secondarily to thrombotic occlusion of the superior mesenteric artery (SMA) is a relatively rare condition. This is due to the fact that the SMA and the celiac trunk are interconnected, and, in a majority of patients, both of these arteries should be significantly narrowed to be symptomatic. Thrombosis-triggered ABI is primarily found in elderly patients. It is typically preceded by abdominal symptoms, such as postprandial pain, weight loss and/or abstaining from food. The diagnostic workout in these symptomatic patients usually reveals atherosclerotic and often highly calcified plaques in the proximal parts of the visceral arteries. In some patients these plaques extend to the adjacent abdominal aorta. A decompensation of the arterial flow in the SMA, resulting in complete thrombotic occlusion of this artery, typically follows hemorrhages, significant dehydration, surgical procedures or cardiac failure events [1,2,3].

The resulting ABI is a life-threatening emergency. An emergency open surgical repair is still the main and recommended treatment modality for ABI. However, considering the high periprocedural morbidity and mortality, endovascular and hybrid approaches have also been suggested as an alternative option. A hybrid revascularization consists of open surgical laparotomy, which allows for an inspection of the intestines, and retrograde endovascular revascularization of the SMA, through direct puncturing of this artery. However, such a hybrid procedure requires general anesthesia, which is not always a valid option in critically ill patients. Therefore, percutaneous endovascular revascularization of the SMA for the treatment of ABI should always be considered.

With advances in endovascular techniques and armamentarium, these less invasive methods have gained more attention [4,5,6,7,8,9,10]. Although the consensus document by the European Society of Vascular Surgery recommends an endovascular approach as the first line therapy (class of recommendation IIa, level of evidence B) [6], recent meta-analysis did not reveal significant differences between endovascular and surgical revascularizations in terms of early and late clinical outcomes [11]. It should, however, be noted that a majority of papers on ABI describe results of the treatment of patients presenting with both embolic and thrombotic occlusions. There are relevant differences between ABI patients with embolic vs. thrombotic etiologies, regarding clinical picture, mortality and the feasibility of transcutaneous revascularization.

A majority of endovascular revascularizations for thrombotic occlusions of the SMA, especially if the angle between the aorta and the SMA is favorable, and the most proximal part of the SMA is patent, are not very challenging and can be performed even by less experienced interventionalists. Still, in some patients presenting with ABI, these procedures can be very difficult and prone to intraprocedural complications. During endovascular reconstruction, on the one hand a full revascularization of ischemic bowel should be achieved. On the other hand, however, the procedure should not be too aggressive, in order to minimize the risk of very dangerous complications, including the rupture or dissection of the aortic wall.

All these challenges particularly concern patients with a completely occluded proximal part of the SMA, where navigation to the distal part of this artery from the aorta is not technically feasible, and also patients presenting with highly calcified plaques in the SMA that extend to the aortic wall, which makes a typical angioplasty of the SMA dangerous, since the high-pressure balloons that should be used to address such lesions can dissect the aortic wall.

In this paper we discuss technical aspects of these difficult endovascular interventions, which are exemplified by the details of endovascular management of an ABI patient who presented with highly calcified plaques in the SMA extending to the aortic wall. In order to avoid a risky high-pressure balloon angioplasty, we applied the lithotripsy-assisted angioplasty.

## 2. Case Report—Calcified Plaques in the SMA Extending to the Aortic Wall

**Figure 1:**
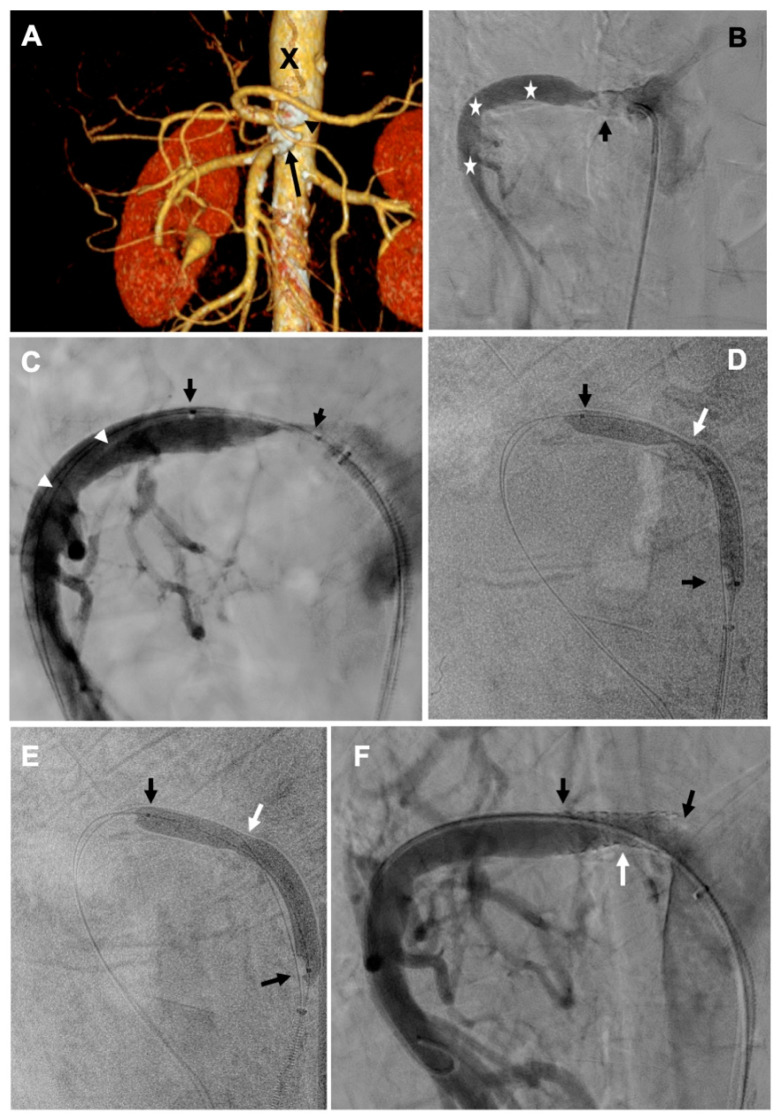
Consecutive steps of revascularization of a narrowed superior mesenteric artery with the use of lithotripsy-assisted angioplasty. (**A**) CT angiography of the aorta (X) and visceral arteries: massive calcifications in the area of the celiac trunk orifice (arrowhead) and the superior mesenteric artery (arrow); (**B**) angiography of the superior mesenteric artery (asterisks)—the superior mesenteric artery with critical stenosis in the proximal part and massive calcifications within this lesion (arrow); (**C**) angiographic result after predilatation with the 3.5/20 and 4.5/20 balloons (balloon between 2 black arrows); balloon angioplasty failed to improve the lesion, and there was >80% residual stenosis; (**D**) balloon lithotripsy system 6.0/60 Shockwave M5+ (between two black arrows)—before dilation using the lithotripsy system; (**E**) balloon lithotripsy system 6.0/60 Shockwave M5+ (between two black arrows)—after application of sonic energy and balloon angioplasty: almost complete dilatation of the lesion; (**F**) final angiographic result: stent visible between two black arrows with a small <5% residual lesion (white arrow; locations of this lesion in subfigures (**D**,**E**) are also marked with white arrows) and good inflow to the distal branches.

A 72-year-old male patient presented with clinical signs of bowel ischemia. He had a history of myocardial infarction and ischemic stroke of the left cerebral hemisphere. He complained of abdominal pains, although there was still no weight loss. This patient was admitted to our hospital due to significant worsening of his abdominal symptoms, which suggested ABI. CT angiography revealed almost complete occlusions of the celiac trunk and the SMA. Moreover, there were massive calcified plaques in this area, which made a precise assessment of the visceral arteries nearly impossible (Figure 1A). Biochemical laboratory assessment demonstrated a slightly elevated lactate, significantly increased troponin and normal CK-MB. Of note, there were neither clinical nor ECG signs of myocardial ischemia. Since all these tests and clinical signs were indicative of ABI, while the patient presented with a high risk of surgical laparotomy, the decision, upon an endovascular intervention, was made. Considering angioarchitecture of the SMA and the celiac trunk, as revealed by CT angiography in this patient, particularly regarding the angle between the aorta and the SMA, femoral access was chosen.

Catheter angiography of the celiac trunk revealed an unfavorable anatomical variant, with a large splenic artery and very narrow remaining branches. Selective catheterization of the SMA demonstrated critical stenosis of its proximal part (Figure 1B), yet there was still a thin lumen left in this artery. However, a standard navigation to the distal segments of the SMA, due to calcified plaques that critically narrowed this artery in its proximal segment, was not possible. Therefore, using the telescoping technique, we advanced the 6F Destination Guiding Sheath (Terumo, Tokyo, Japan) and the RBI diagnostic catheter (Merit Medical Systems, South Jordan, UT, USA) to the ostium of the SMA. Then, using the buddy wire technique, we navigated across the stenosis with two 0.014” guidewires. Finally, we performed three inflations under a pressure of 10–16 atm. with the 3.5/20 mm and 4.5/20 mm TREK™ Coronary Dilatation Catheters (Abbott Vascular, Abbott Park, IL, USA). After this balloon angioplasty, the lesion minimally improved (Figure 1C). Since the use of a larger high-pressure balloon seemed to be associated with considerable risk of aortic wall rupture, considering the calcified characteristics of the plaques, we decided upon the use of a lithotripsy device to address this lesion. We explained the treatment strategy to the patient, and he gave his informed consent. We introduced the 6.0/60 mm Shockwave M5+ intravascular lithotripsy catheter (Shockwave Medical, Santa Clara, CA, USA) to the proximal part of the SMA (Figure 1D) and at the level of calcified plaque, which significantly narrowed the proximal segment of this artery, we inflated the balloon of this system under a pressure of 2–4 atm. and performed four applications of sonic energy, which resulted in dilatation of the lesion (Figure 1E). Then, inflating the balloon under a pressure of 14 atm., we implanted the 7 × 19 mm Omnilink balloon-expandable stent (Abbott Vascular, Abbott Park, IL, USA), achieving an almost complete dilatation of the artery and good inflow to the periphery (Figure 1F). The total radiation dose associated with this reconstruction was 1.9 Gy, while the total volume of contrast used during the procedure was 80 mL (in this patient, the previously performed CT angiography provided quite a good mapping, which allowed for reduction in the amount of contrast used).

After stent implantation, a residual stenosis was at the level of 5%. In the postprocedural course the patient no longer complained of abdominal symptoms. Although after the procedure there was a temporary increase in serum lactate (from 1.2 to 1.5 mmol/L) and troponin (to 183 g/mL)—but with normal CK-MB—the following biochemical markers normalized: lactate in the first postprocedural day and troponin in the forth. There were neither clinical symptoms of intestinal ischemia nor other adverse events during 6 months follow-up.

## 3. Discussion

As has already been described in the introduction, endovascular revascularization of the SMA in patients presenting with ABI becomes a valid alternative to open surgical repair or hybrid procedures. However, while revascularization of the SMA occluded by emboli is less challenging, and can be addressed by aspiration embolectomy or other less technically demanding procedures, thrombotic occlusions of the SMA, particularly in patients presenting with significant comorbidities, can be very difficult. Optimally, the patent proximal segment of the SMA should be at least 5–10 mm long. In addition, a sharp angle between the aorta and the SMA, particularly when patent segment of the SMA is short (less than 5 mm), makes the stabilization of the guiding sheaths and catheters difficult; in such cases, brachial or radial access is preferred to femoral access.

The especially challenging pathologies associated ABI comprise the following: a complete occlusion of the SMA, and highly calcified plaques in the SMA extending to the aortic wall. In the first situation, when a direct navigation to the occluded proximal segment cannot be performed from the aortic side, a retrograde revascularization should be performed [12,13,14,15,16]. Such a retrograde revascularization should be considered when the intraprocedural catheter angiography fails to reveal the origin of the SMA. Navigation to the distal part of the SMA can either be performed through the branches of the celiac trunk [12,13,14,15] or through the inferior mesenteric artery [16]. The pre- and intraprocedural diagnostic workout should demonstrate which of these two potential routes is feasible and will be easier to perform. Currently available endovascular armamentarium allows for safe navigation through the branches of the celiac trunk or the inferior mesenteric artery, on the condition that the interventionalist is aware of the anatomy of this vascular territory, including anatomical variants that are quite prevalent [17], and is experienced in the use of tiny and flexible catheters and guidewires. Particularly, the anatomy of the connections between the SMA and the celiac trunk or the inferior mesenteric artery should be acknowledged. In some individuals, these connections, which typically are present alongside the superior and inferior pancreaticododenal arteries, do not exist or differ significantly from the “textbook” anatomy.

During retrograde revascularization of the SMA, there are several potential difficulties that need to be addressed. The first one concerns navigation through tiny and fragile arteries of collateral circulation. Then, the guidewire should be properly placed in the proximity of the occluding lesion, and an optimal place where the recanalization should be initiated should be determined. Navigation with the guidewire across the occluding plaque into the aorta is particularly challenging. A situation when the guidewire crosses the plaque but then drifts and subinimally penetrates the aortic wall is not so rare. The interventionalist should be aware of such a possible drift, and the proper position of the guidewire should be confirmed in several radiological views, since a standard one may not reveal this dangerous displacement. A misdiagnosis of the above-described subintimal penetration of the aortic wall can result in its rupture during subsequent steps of the reconstruction, which would require implantation of an aortic stent graft. Of note, this retrograde navigation across the occluding plaque should solely be performed with a guidewire, without supporting guiding sheath, since the arteries of the collateral circulation are narrow and fragile, and even low-profile catheters cannot be safely used here. It should also be remembered that after successful revascularization and stenting of the SMA, the microcatheter placed in the collateral circulation should be left there, the guidewire should be removed first, and then, after a final check of the intestinal circulation, the microcatheter should be removed.

Regarding highly calcified plaques in the ostium of the SMA, the risk of aortic wall dissection during forceful dilatation with high-pressure balloons should be considered. Such an injury of the aortic wall at the level of the celiac trunk and the SMA is very difficult to repair, both surgically as well as endovascularly. Lithotripsy offers a valuable alternative, since it destabilizes the calcified lesion, while the procedure is performed under relatively low pressure. Even if lithotripsy is not free from potential complications, it is nonetheless safer than traditional balloon angioplasty and allows for subsequent dilatation with an angioplastic balloon.

Recently, intravascular lithotripsy has been seen as a useful and safe method for endovascular management of highly calcified atherosclerotic plaques in such arterial territories as the coronary, carotid, mesenteric and renal arteries, as well as arteries of the extremities. However, currently available lithotripsy systems require the use of a 0.014” guidewire, which is associated with a potential instability of the entire system. The buddy wire technique, which was used in our patient, can solve this problem. This particular endovascular technique utilized an additional parallel guidewire, positioned alongside the primary one, to improve the support, steerability, and trackability of the balloons and catheters. The lithotripsy-assisted angioplasty is a relatively new endovascular method, but has already been reported to be safe and useful for addressing calcified lesions in the coronary, carotid, renal and lower limb arteries [18,19,20,21,22,23,24,25]. It seems that this technique can also be used for the recanalization of highly calcified SMA lesions, which was demonstrated in this case. However, in this particular patient, there was still a residual stenosis, most likely of negligible clinical relevance. This minor stenosis was probably associated with the conservative approach to the potentially dangerous plaque; the procedure was not aimed at ideal, but rather hemodynamically satisfactory, recanalization. In addition, considering the highly calcified characteristics of the lesion and adjacent aortic wall, it was probably not possible to press the plaque completely into the vascular wall.

Of note, such endovascular reconstructions of the visceral arteries are typically associated with relatively high radiation doses (in the above-discussed patient it was 1.9 Gy), which usually results from long procedure times and the need for the left anterior oblique (LAO) radiological view for better visualization of the target arteries. This particular radiological view is known to be associated with high radiation doses. Therefore, a proper shielding is of particular importance during these revascularizations.

It should be emphasized that the above-described endovascular techniques, as of yet, are not the standard ones. Yet, in this paper, we present how technical challenges associated with the difficult-to-manage occlusions of the SMA can be overcome, and how endovascular devices, which have an already-established role in other arteries, can be used in this blood vessel. We hope that, until endovascular revascularization for ABI becomes a standard procedure, our paper may be valuable for interventionalists and will offer “tips and tricks” to manage the most challenging patients presenting with this life-threatening disease.

## Data Availability

The data presented in this study are available on request from the corresponding author, still restrictions may apply in order to protect patient’s privacy.
